# Transitional Experiences of Internationally Qualified Midwives Practicing in Australia: Protocol for a Mixed Methods Study

**DOI:** 10.2196/13406

**Published:** 2019-06-01

**Authors:** Mitra Javanmard, Mary Steen, Rachael Vernon, Megan Cooper

**Affiliations:** 1 School of Nursing and Midwifery Division of Health Sciences University of South Australia Adelaide Australia

**Keywords:** internationally qualified midwives, experiences, views, adjustment, transition, integration

## Abstract

**Background:**

Approximately 13% of the total Australian midwifery workforce is internationally qualified. Although the internationally qualified midwives (IQMs) play a significant role in the Australian midwifery system, there is limited understanding of their transitional experiences.

**Objective:**

The objective of this study protocol is to explore the transitional experiences and views of IQMs practicing in Australia, through the investigation of demographic profiles and key challenges that influence a smooth transition.

**Methods:**

This paper presents an explanatory sequential mixed methods study protocol. This protocol incorporates an e-survey and individual interviews. The e-survey in the first phase will be distributed to IQMs in Australia via the website e-bulletins of the Australian Nursing and Midwifery Federation and the Australian College of Midwives. Additionally, potential respondents will be recruited via social media (ie, Twitter and Facebook) and associated snowball sampling. Data from the e-survey will be statistically analyzed. At the end of the e-survey, respondents will be asked whether they are willing to take part in an interview. The results of the e-survey and relevant literature review will help to develop a guideline for interview questions for the second phase. In phase two, a purposeful sample of participants will be recruited using the same selection criteria as for the e-survey. Semistructured interviews will provide a deeper insight into the transitional experiences of IQMs. Data from the interviews will then be thematically analyzed.

**Results:**

An integration of the e-survey results (phase one) and interview findings (phase two) will be synthesized to explore and better understand the transitional experiences of this group of midwives. It is anticipated that data collection and analysis will be completed by June 2019 and results will be disseminated through peer-reviewed publications in late 2019.

**Conclusions:**

This research protocol may generate new knowledge about the transition of IQMs in Australia. These findings could be used to formulate recommendations to inform the transition of future IQMs in Australia.

**International Registered Report Identifier (IRRID):**

DERR1-10.2196/13406

## Introduction

### Background

Australia is a multicultural country with 49% of the Australian population (24.77 million) either born overseas or having at least one parent who was born overseas [1]. As in many other fields, multiculturalism affects the health professional workforce [2]. For example, the 4244 internationally qualified midwives (IQMs) who received their midwifery qualification in countries other than Australia and are currently practicing in Australia represent 12.99% of all registered midwives (4244/32,669) in the country [3]. Globally, there has been increasing reliance on IQMs and internationally qualified nurses (IQNs) within the health workforce of some developed countries [4-6]. A shortage of nurses and midwives, coupled with increased demands for health care, may be two of the factors driving this increase [7,8]. However, the transition of this group of health professionals into the Australian health care system has been accompanied by various challenges [8-11]. Pilette [12] explains that the process of adjustment into a foreign health care system may take about one year and is comprised of four different phases, including acquaintance, indignation, conflict resolution, and integration. He noted that each phase is associated with unique challenges [12]. Furthermore, it is possible that IQNs from culturally and linguistically diverse backgrounds may find it takes longer to adjust [13].

Differences in nursing practices, along with a lack of familiarity with local technologies, policies, and guidelines, are reported as major challenges for IQNs [11,14,15]. IQNs from different educational backgrounds may face stressful situations due to different ways of undertaking clinical procedures [16], which can result in frustration and anxiety [17]. Deskilling and lack of recognition of IQNs’ capabilities, skills, and experiences can cultivate feelings of invisibility and marginalization, which may have a negative effect on self-esteem, confidence, and well-being [14,18]. These factors are important to consider and acknowledge as these may be similar for IQMs. The literature also highlighted multidimensional discrimination and cultural impositions experienced by IQNs in some health care systems of destination countries [11,15]. Diversity in race, color, culture, or language can be a trigger of inequality of opportunities and racism in the form of bullying by staff or rejection of care by some patients [13]. For IQMs and IQNs, racial discrimination can lead to intimidation, public humiliation, social exclusion, and loss of confidence and professional authority [6,15,19].

Proceeding on the basis of Cooper's Taxonomy [20], a structured literature search was conducted by the authors [21]. The five steps that guided the literature review included *formulate the problem*, *search the literature and gather information*, *evaluate study quality*, *analyze*, and *interpret the data* [20]. Only two studies reported the experiences of midwives practicing in a foreign country; one study examined UK midwives’ experiences of working in Australia [6] and another in New Zealand [10]. The literature appears to focus mainly on internationally qualified nurses and doctors, with midwives usually included within the broader category of nurse migration. However, the International Confederation of Midwives has stated that “midwifery should be recognised as an autonomous profession globally” [22].

Although midwifery is an internationally mobile profession, there appears to be a global gap in literature that explores the transition experiences of IQMs moving between countries [23]. Hence, this lack of research, as well as the need to investigate and explore the transitional experiences of IQMs as they transition into Australian maternity services, is clear justification for undertaking this doctoral study. Understanding the transitional experiences of this group of midwives into the Australian midwifery system, as well as disseminating the findings, will increase awareness of challenges that IQMs may experience.

### Study Aim and Objectives

Given the identified gap in the literature, this study aims to explore the transitional experiences and views of IQMs practicing in Australia. The objectives of this study are to investigate demographic profiles of IQMs in Australia and to explore the key challenges that promote or hinder a smooth transition into this workforce. The research questions for this study are as follows:

1. What are the demographic characteristics of IQMs practicing midwifery in Australia?
2. What challenges do IQMs face during their transitional process into the Australian midwifery workforce?
3. How do IQMs practicing midwifery in Australia perceive the level of:
  - peer support?
  - peer respect?
  - peer acceptance?

## Methods

### Study Design

The researchers will use an explanatory, sequential, mixed methods design, incorporating two phases to provide valid and credible outcomes [24]. This mixed methods design is underpinned by the philosophical assumptions of *pragmatism*, which guides both phases [24-26]. The research emphasizes the use of the pluralistic approaches in order to achieve a deeper understanding of the transitional experiences of individual IQMs, as well as the challenges they confront during their transition into the Australian midwifery workforce [24-27]. Regarding pragmatism, the criterion of “what works?” will be used to select the best methodologies to address the research questions and, ultimately, to find some strategies to solve any challenges, as required [26].

This research protocol incorporates an e-survey and individual interviews. Phase one includes an e-survey study that is currently being undertaken to capture IQMs’ demographic profiles, their transitional experiences, and positive and negative factors contributing to their transition during the first year of their practice in Australia. The exploratory nature of the second phase requires a qualitative approach to identify and describe the transitional experiences and views of individual IQMs. Hence, qualitative descriptive design based on semistructured interviews will be used for phase two to provide a detailed description of the experiences and views of IQMs in language similar to their own forms of expression [28,29].

The results from the e-survey will assist in determining questions for the interviews to explore, clarify, and affirm the experiences and perspectives of IQMs [26]. The findings of the interviews will provide further explanations for unexpected results that may emerge during the e-survey study [24-26]. The final inferences will be presented on the basis of integrated data from both phases of the study [27].

### Study Population

The target population for this study is a sample of midwives who obtained their midwifery qualifications outside of Australia, who practiced for a minimum of 12 months in the countries in which they obtained their midwifery qualifications, and who are presently practicing as midwives in Australia. In order to maximize recruitment opportunities, a specific length of time of employment in Australia will not specified. It is beyond the scope of this research to consider the experiences of midwives who obtained their midwifery qualifications within Australia.

### Data Collection and Setting

IQMs will be invited to participate in this study nationally within Australia.

#### E-Survey

IQMs practicing in Australia are being accessed via a nonprobability sample design, which also incorporates factors such as convenience and snowball sampling. The Nursing and Midwifery Board of Australia was approached at the beginning of respondent recruitment, with a request made to access the contact details of all registered IQMs in Australia. This request was denied due to the Board’s privacy policy. There are no other official organizations or bodies that collect contact details of IQMs in Australia. A nonprobability sampling framework and an opt-in e-survey are being used, as a random sample cannot be accessed.

As such, the most effective method to recruit as many IQM respondents as possible is via the Australian Nursing and Midwifery Federation and Australian College of Midwives websites. The e-bulletins of these midwifery professional bodies are advertising and distributing the e-survey, which is linked to SurveyMonkey, a Web-based survey platform. Other approaches being used to recruit potential respondents include snowball sampling and social media platforms, such as Facebook and Twitter.

Calculation of the potential sample size is not feasible for this e-survey study, due to using a nonprobability sampling framework and undertaking an opt-in e-survey, and this is acknowledged as a current limitation of the study. Furthermore, a response rate cannot be calculated due to the inability to identify the contact details of IQMs and the associated absence of a sampling frame [30,31]. Finally, a power calculation is not appropriate for this descriptive e-survey study, as a hypothesis is not being tested [32].

The data collection tool was developed by adapting pre-existing and relevant questionnaires used by Giegerich [33] and the Australian Midwifery Workforce Survey [34].

The adapted descriptive questionnaire is comprised of the following elements:

1. A total of 26 closed-ended questions designed to collect IQMs’ demographic characteristics and their current working arrangements in Australia.
2. A total of 12 7-point Likert-scale questions to capture the multidimensional perspectives of the IQMs’ transitioning experiences during their first 12 months of working in Australia.
3. Two open-ended questions to offer IQMs the opportunity to further share their experiences.

Prior to administration of the e-survey, the adapted questionnaire was assessed for content and face validity by a panel of three experts who had survey development expertise or experience of working with IQMs in Australia. The e-survey is hosted via SurveyMonkey and presented over 10 pages; it will allow respondents to review and change their answers prior to completion. Once the e-survey has been completed, respondents will not be able to change their responses nor will they be able to complete the e-survey again. The e-survey will be administered in English; the estimated time to complete the questionnaire is approximately 15-20 minutes (see Multimedia Appendix 1).

#### Interviews

The consolidated criteria for reporting qualitative studies (COREQ), a 32-item checklist, will be followed in this phase to ensure all aspects of the study methods, analysis, findings, and interpretations are considered [35]. Participants will be recruited nationally within Australia. To recruit participants, a purposeful sample using the same selection criteria as for the e-survey study will be used [36]. Therefore, the sample will be a subset of the e-survey study. At the end of the e-survey, respondents will be asked whether they are willing to take part in an interview and, if so, they will be encouraged to contact the primary researcher voluntarily.

The interview guidelines will be developed based on the e-survey results and relevant literature and will then be reviewed by all authors, three of whom are experienced researchers (MS, RV, and MC). Questions will be open-ended with a broad focus to explore the transitional experiences of IQMs. Semistructured interviews—face-to-face or telephone interviews—will be digitally audio recorded with the prior consent of the participants and each will last approximately 30-45 minutes. All interviews will be professionally transcribed for analysis by using a consistent template to obtain consistency between the transcripts.

Although we are not able to specify the sample size needed to reach data saturation, it is estimated that we will need to interview approximately 6-12 participants, based on evidence published by Guest et al [37]; however, it may depend on the richness and depth of gathered data [38]. Data collection will continue until saturation is achieved: that is, when we no longer identify new data or when confirming or disconfirming data can be reached with respect to the research questions [37-39]. Transcripts will be returned to all participants and they will be invited to review them for accuracy.

### Data Analysis

#### E-Survey

The aim of this e-survey study is to provide descriptive results for the research questions by analyzing the e-survey responses [40]. All responses will be analyzed even where they are incomplete. Statistical analysis will be conducted using SPSS Statistics for Windows, version 22.0 (IBM Corp). Descriptive statistics will be used to determine sample characteristics, such as frequencies, percentages, means, and standard deviations of the total sample of ordinal outcomes [26]. Ordinal outcomes from Likert scales will be analyzed using the nonparametric Mann-Whitney U test to compare the transitioning experiences of IQMs from English-speaking backgrounds and non-English-speaking backgrounds, if required. Statistical significance will be identified as *P*<.05, if necessitated.

Qualitative analysis of the two open-ended questions in the e-survey will be conducted manually. Summative content analysis [41] will be used to identify categories that best describe the respondents’ views. This will involve counting of common words or phrases to analyze similarities and differences between participants’ responses [41]. Significant phrases will be identified and findings will be formulated. The final analysis will be agreed upon by consensus to assure consistency and confirmation of results.

#### Interviews

Interviews will be analyzed thematically, following the six-step approach explained by Braun and Clarke [42]. This systematic approach includes data familiarization, generation of initial codes, thematic search, thematic review, thematic deﬁnition, and reporting. Analysis will commence by first entering the transcribed interviews into a Microsoft Excel worksheet to manage and summarize the corpus of data and to generate columns consisting of all comments. Multiple readings of the transcripts will be undertaken to understand participants’ meanings and to find similarities and differences of their experiences. Words or phrases relevant to the research questions will be coded and relevant data will be collated to each code. Codes will then be sorted into potential themes, while the relevant coded data extracts will be collated within these identified themes. Once coding is complete, all researchers will compare findings. Codes that express similar meanings will be highlighted with the same color. The concepts and categories will be reviewed multiple times to ensure a coherent pattern. Following this, the validity of individual themes will be considered. Finally, as part of the refinement process, themes containing subthemes will be identified. The final analysis will be agreed upon by consensus to assure consistency and confirmation of findings.

## Results

### Overview

Findings from the e-survey study and interviews will be drawn together and compared in order to synthesize the final results and to reach an overall interpretation. To integrate data, *triangulation protocol* will be followed, a technique that has been explained by O'Cathain et al [43]. For this technique, findings from each phase of the study will be listed on the same page, helping to identify findings that are potentially convergent, complementary, or discrepant. In order to interpret the data from a multidimensional perspective, the datasets will first be separately analyzed deductively and inductively; we will then move back and forth between the datasets, with knowledge produced by each one, and will finally bring them together [44]. It is anticipated that data collection and analysis will be completed by June 2019 and results will be disseminated through peer-reviewed publications in late 2019.

### Rigor

To promote integrity and quality in this mixed methods study, appropriate strategies will be applied during each phase of the study as follows [24,26]. Regarding the e-survey, content and face validity were conducted for the adapted questionnaire prior to administration of the e-survey. Regarding the interviews, credibility, transferability, dependability, and confirmability will be considered to ensure trustworthiness of the study [45,46]. Consistent use of interview guidelines will ensure consistent data collection [39]. Participants' views will be accurately reflected via the use of audio recording; to maintain consistency between the transcripts, a consistent template will be used for each transcript [35]. Moreover, participant validation will occur in which participants will review and verify the interpretations [47]. Confirmability will be ensured through the use of an audit trail, where the activities exercised in the study, both in the collection of data and its analysis, will be accurately detailed and recorded [46]. Furthermore, the researchers will achieve confirmability through the use of participant quotes to support the findings. A reflexive process will be documented to reduce subjectivity bias. This will be supported by a clear audit trait of decision making throughout the study [48]. Publication and presentation of findings for target readers, such as the study participants, researchers, and other IQMs, will provide opportunities to enhance credibility [46]. In addition, a mixed methods design has been employed for this study, which will enable data triangulation to take place [24-26].

### Ethical Considerations

This study has obtained ethical approval from the University of South Australia Human Research Ethics Committee on July 4, 2017 (protocol number: 0000036397). All information about the nature and aim of the study, confidentiality, anonymity, voluntary nature of participation and withdrawal, potential benefits and risks of the study, contact details of a person designated to receive complaints, and contact details of the research team will be provided to participants of this study, according to ethical guidelines [49,50]. Consent to participate in the e-survey is implied by the respondent’s voluntary completion of the SurveyMonkey questionnaire. Written consent will be obtained from all participants who agree to be involved in phase two interviews. Phase one responses will be collected anonymously in SurveyMonkey. Participants who agree to be involved in phase two will be interviewed, either face-to-face or by telephone, at a mutually convenient date, time, and place. All data collected will be stored securely for 5 years. Participants will not receive any incentive for agreeing to be involved in this study.

## Discussion

Motivational factors underpinning the migration of internationally qualified health professionals (IQHPs) to developed nations have been highlighted in the literature, with these factors including the desire for an increased standard of living, higher education, professional experiences, and opportunities to achieve better pay [4,8,17]. However, the literature also highlights contradictions between the expectations of IQHPs before commencing work in their host countries and their actual experiences [13,18].

Migrating to a new country with differences in midwifery and nursing practices, different workplace cultures, and the presence of bullying and discrimination may be confronting for IQMs and IQNs. This could create further challenges during the process of their integration into a foreign midwifery and nursing clinical workforce [21]. Different educational backgrounds, different ways of undertaking clinical procedures, and different guidelines and policies may negatively influence the adjustment of IQMs and IQNs into a new health care workforce [16]. Consequently, the experience of such stressful situations may lead to frustration and anxiety [17]. Lack of recognition of the capabilities, skills, and experiences of IQNs may result in feelings of invisibility and marginalization. This, in turn, may negatively affect their confidence, self-esteem, and well-being [14,18,51,52].

An intrinsic barrier for IQNs attempting to adjust to a new health care system is a lack of familiarity with the culture of local health practice [14,16,52-54]. The literature broadly discusses the culture clash experienced due to feelings of not fitting in and isolation [52-54]. Moreover, migrants from culturally and linguistically diverse backgrounds can find these challenges more problematic [55].

The literature highlights that IQNs face cultural impositions and multidimensional discrimination in some health care systems of their host countries [11,15,56] and, markedly, that bullying may stem from racism towards IQNs and IQMs [21]. Racial discrimination may give rise to intimidation, social exclusion, and public humiliation, resulting in IQMs and IQNs experiencing a loss of professional authority [6,15,19,57].

The process of migrating to Australia and working as a midwife can be a complex and challenging one [14,58]. To gain insight into how to best support the needs of IQMs, such challenges need to be explored and understood [13,18]. Midwives have long migrated and worked across borders, but their needs and perspectives have been paid relatively minimal attention, especially during their adjustment into the Australian midwifery workforce. With a prediction of continuing recruitment of IQMs and the lack of studies undertaken on this cohort, the challenges of their professional integration need to be explored. This is an area essential to advancing research and practice. Currently the first of its kind in Australia, this study protocol has been designed to address this priority through exploring the experiences and views of IQMs practicing in Australia. The insight gained from the understanding of IQMs’ enablers, as well as their barriers, may be of great value.

## Appendix

Multimedia Appendix 1 The adapted questionnaire.

## Abbreviations

COREQ: consolidated criteria for reporting qualitative studies
IQHP: internationally qualified health professional
IQM: internationally qualified midwife
IQN: internationally qualified nurse
