# Supplementary material for: Transitional Experiences of Internationally Qualified Midwives Practicing in Australia: Protocol for a Mixed Methods Study
Source: JMIR Res Protoc. 2019 Jun 1;8(6):e13406. doi: 10.2196/13406 (PMC6592485; doi:10.2196/13406)
Supplement: Multimedia Appendix 1 [file resprot_v8i6e13406_app1.pdf]

Questionnaire (E-survey)

A survey of experiences of internationally qualified midwives in Australia.

Demographics:

1. What is your gender?

- Female
- Male

2. What is your age?

- 20-25
- 26-30
- 31-35
- 36-40
- 41-45
- 46-50
- 51-55
- 55 or more
- I would prefer not to answer.

3. What is your country of birth? ………

4. Which of the following best describes your relationship status on moving to Australia?

- Single, never married
- Married/De facto
- Separated
- Divorced
- Widowed
- In a domestic partnership or civil union
- Single, but cohabiting with a significant other
- I would prefer not to answer.

5. In what country did you qualify as a midwife? …….

6. In what language was your midwifery education taught?

- ○ English
- ○ Chinese
- ○ Italian
- ○ Greek
- ○ Arabic
- ○ Hindi
- ○ Spanish
- ○ Other (Please specify) ……

7. In what year did you qualify as a midwife? …..

- ○ 2017
- ○ 2016
- ○ 2015
- ○ 2014
- ○ 2013
- ○ 2012
- ○ 2011
- ○ 2010
- ○ 2009
- ○ 2008
- ○ 2007
- ○ 2006
- ○ 2005
- ○ 2004
- ○ 2003
- ○ 2002
- ○ 2001
- ○ 2000
- ○ 1999
- ○ 1998
- ○ 1997
- ○ 1996
- ○ 1995

- 1994
- 1993
- 1992
- 1991
- 1990
- 1989
- 1988
- 1987
- 1986
- 1985
- 1984
- 1983
- 1982
- 1981
- 1980
- 1979
- 1978
- 1977
- 1976
- 1975
- 1974
- 1973
- 1972
- 1971

8. What is your initial midwifery qualification? (Please select one option)

- Hospital/Certificate
- Diploma
- Bachelor’s Degree
- Other (Please specify) ……

9. What is the highest academic qualification you hold? (Please select one option)

- Bachelor’s Degree
- Bachelor’s Degree (Honours)

- Postgraduate Certificate
- Postgraduate Diploma
- Master’s Degree
- Professional Doctorate
- Doctor of Philosophy (PHD)
- None of the above apply

10. How many years did you practice as a midwife in your country of midwifery registration?

- Up to 1
- 2
- 3
- 4
- 5
- 6
- 7
- 8
- 9
- 10 or more

11. Please list the countries you worked as a midwife outside of your country of midwifery registration prior to Australia. ……….

12. I was motivated to migrate from my country of midwifery registration to Australia because: (Please check all that apply)

- Lack of available midwifery jobs in my country of midwifery registration.
- Working conditions for midwives were not safe in my country of midwifery registration.
- There were not enough medicines, supplies, and staff to provide effective patient care in my country of midwifery registration.
- Opportunity to make more money from working as a midwife in Australia.
- Having additional opportunities for midwifery education in Australia.
- Several family members had already moved to Australia.
- I would prefer not to answer
- Other (Please specify) ………

13. Please rank and order (most important to least important factors) that influenced on your decision to migrate to Australia.

- ○ Personal Reasons (To travel/experience a different way of living)
- ○ Professional Reasons (To gain professional development/higher education)
- ○ Financial Reasons (To earn more money from working as a midwife in Australia/to be able to send money home)
- ○ Social Reasons (To join family and/or friends in Australia/to enable children to grow up in Australia)

14. What year did you migrate to Australia?

- ○ 2017
- ○ 2016
- ○ 2015
- ○ 2014
- ○ 2013
- ○ 2012
- ○ 2011
- ○ 2010
- ○ 2009
- ○ 2008
- ○ 2007
- ○ 2006
- ○ 2005
- ○ 2004
- ○ 2003
- ○ 2002
- ○ 2001
- ○ 2000

  …….

15. What year did you begin working as a midwife in Australia?

- ○ 2017
- ○ 2016
- ○ 2015

- 2014
- 2013
- 2012
- 2011
- 2010
- 2009
- 2008
- 2007
- 2006
- 2005

…….

16. How many years have you practised as a midwife in Australia?

- Up to 1
- 2
- 3
- 4
- 5
- 6
- 7
- 8
- 9
- 10 or more

17. What type of English language test did you sit to obtain your midwifery registration in Australia?

- IELTS (International English language Testing System)
- OET (Occupational English Test)
- Not applicable
- Other (Please specify) …….

18. Which of the following methods did you use to find your first job as a registered midwife in Australia? (Please check all that apply)

- Someone you knew worked at the hospital or an agency
- Public advertisements, job listings, or internet sources

- Advice from a family member
- Advice from faculty/teachers in pre-registration course, if you had one
- Australian midwife recruiter or hospital placement service
- Other (Please specify) …….

19. Did you undertake any of the following options to prepare you for registration as a midwife in Australia? (Please check all that apply)

- Preparation/pre-registration programs
- Clinical placement
- English classes
- Cultural information
- Technology training
- Specific information about the Australian healthcare system
- Not applicable
- Other (please specify) …….

20. Did these options help you have a smooth transition into the Australian midwifery workforce?

- Yes
- No
- Not applicable

21. Please explain your response to the above question? ………

Transitional Experiences:

22. Which healthcare sector did you start working in Australia?

- Public healthcare sector
- Private healthcare sector

23. Please respond to the following questions related to your transition experiences as a registered midwife during your first 12 months in Australia. Please select the answer that best reflects your experiences.

	Strongly Agree	Agree	Somewhat agree	Neither agree or disagree	Somewhat disagree	Disagree	Strongly disagree
I had a satisfactory orientation period in my workplace when I started working as a midwife in Australia.	○	○	○	○	○	○	○
The orientation I received at my workplace prepared me for working independently.	○	○	○	○	○	○	○
When I first started working as a midwife in Australia, I became aware of the differences between the Australian culture and my own culture.	○	○	○	○	○	○	○
When I first started working as a midwife in Australia, I noticed the differences between Australian midwifery care and midwifery care in my country of midwifery registration.	○	○	○	○	○	○	○
When I had questions, I knew who to ask or where to get the answer.	○	○	○	○	○	○	○
I experienced some challenges in understanding professional/medical language or ideas in the English language.	○	○	○	○	○	○	○
I experienced language challenges when communicating with colleagues and women I cared for.	○	○	○	○	○	○	○
I felt I was respected as a midwife by the women I cared for and their families in Australia.	○	○	○	○	○	○	○
I felt I was respected as a midwife by the midwives and doctors I worked with in Australia.	○	○	○	○	○	○	○
I believe my manager fully utilised my skills and experiences.	○	○	○	○	○	○	○
I believe I had the same opportunities for advancement and promotion in my career as my colleagues working in the same organisation.	○	○	○	○	○	○	○
I felt discriminated against while working as a midwife in Australia because of my language skills.	○	○	○	○	○	○	○

24. Which of the following work-related situations were difficult for you because of a language difference? (Please check all that apply)

- ○ Caring for women I cared for
- ○ Working with the families of women I cared for
- ○ Working in emergency situations
- ○ Taking verbal orders from doctors
- ○ Writing, charting, and documenting midwifery care
- ○ Delegating or asking others to assist me with midwifery care, if required
- ○ Talking on the telephone
- ○ Understanding Australian midwifery policies and guidelines
- ○ None
- ○ Other (Please specify) …….

25. Who or what did you find most helpful whilst working as a midwife in Australia? ….

Your current employment situation:

26. Which of the following statements best describes your current employment situation as a midwife?

- ○ Employed and working full time
- ○ Employed and working part time
- ○ Employed and working on casual basis
- ○ Employed, currently on leave
- ○ Self employed
- ○ Unemployed
- ○ Other (Please specify) …….

27. Which of the following most closely describes the principal role of your main job?

- ○ Registered midwife
- ○ Clinical midwife
- ○ Registered nurse and midwife

- Clinical nurse and midwife
- Policy/Administrator
- Educator
- Researcher
- Other (Please specify) ………

28. If employed in a Health Service, in which area do you most commonly work as a midwife?

- Care during labour and birth
- Postnatal care
- Continuum of midwifery care
- Antenatal care
- Neonatal care
- Midwifery management
- Midwifery education
- Midwifery research
- I do not work in a health service
- Other (Please specify) …….

29. Which of the following most closely describes the current work setting of your main job as a midwife?

- Hospital (Excluding outpatient services)
- Community health care services
- Outpatient services
- Tertiary educational facility
- Private midwifery practice
- Other government department or agency
- Aboriginal health services
- Specialist (O&G) practice
- General practitioner (GP) practice
- Other (Please specify) …….

The second stage of the study:

You are invited to participate in the second stage of this study which involves individual interviews that will further explore your experiences in the Australian health care system.

If you are interested in participating in an individual interview, please email me for further information: **mitra.javanmard@mymail.unisa.edu.au** or contact me by telephone: **+61 425 477 771.**

If you would like a copy of the final research report, please contact me by email: **mitra.javanmard@mymail.unisa.edu.au**

Thank you for completing the survey.
